# Outcomes of metastasis-directed therapy of bone oligometastatic prostate cancer

**DOI:** 10.1186/s13014-021-01849-8

**Published:** 2021-06-30

**Authors:** Paul Rogowski, Christian Trapp, Rieke von Bestenbostel, Nina-Sophie Schmidt-Hegemann, Run Shi, Harun Ilhan, Alexander Kretschmer, Christian Stief, Ute Ganswindt, Claus Belka, Minglun Li

**Affiliations:** 1 Department of Radiation Oncology, University Hospital, LMU Munich, Marchioninistr. 15, 81377 Munich, Germany; 2 Department of Nuclear Medicine, University Hospital, LMU Munich, Munich, Germany; 3 Department of Urology, University Hospital, LMU Munich, Munich, Germany; 4 Department of Therapeutic Radiology and Oncology, Innsbruck Medical University, Anichstr. 35, 6020 Innsbruck, Austria; 5 German Cancer Consortium (DKTK), Munich, Germany

**Keywords:** Prostate cancer, Oligometastases, Bone metastases, Radiotherapy, SBRT, Metastasis-directed therapy

## Abstract

**Background:**

The aim of this work was to investigate the outcome of metastasis-directed radiotherapy (MDT) in prostate cancer patients with bone metastases following current ESTRO/EORTC subclassifications for oligometastatic disease.

**Methods:**

Clinical data of 80 consecutive oligometastatic patients with 115 bone lesions receiving MDT between 2011 and 2019 were retrospectively evaluated. Hormone-sensitive (77.5%) and castrate-resistant (22.5%) patients were included. MDT was delivered with conventional fractionated or stereotactic body radiotherapy (SBRT) techniques. Kaplan–Meier method, log rank test, as well as Cox regression were used to calculate local control (LC) and biochemical and clinical progression-free survival (bPFS/cPFS).

**Results:**

At the time of MDT 31% of patients had de-novo synchronous oligometastatic disease, 46% had de-novo metachronous oligorecurrence after primary treatment and 23% had either de-novo oligoprogressive disease, repeat oligometastatic disease or induced oligometastatic disease. The median BED_3_ was 93.3 Gy (range 75.8–95.3 Gy). Concomitant ADT was administered in 69% of patients. After a median follow-up of 23 months the median bPFS and cPFS were 16.5 and 21.5 months, respectively. The 2-year LC rate was 98.3%. In multivariate analysis, age ≤ 70 (HR = 2.60, 95% CI 1.20–5.62, p = 0.015) and concomitant ADT (HR = 0.26, 95% CI 0.12–0.58, p = 0.001) significantly correlated with cPFS. Category of oligometastatic disease and hormone-sensitivity were predictive for cPFS in univariate analysis. Of 45 patients with biochemical relapse, nineteen patients (42.2%) had repeat oligometastatic disease. Fourteen patients (31%) underwent a second course of MDT. No patients experienced grade ≥ 3 toxicities.

**Conclusions:**

MDT is safe and offers high local control rates in bone oligometastases of prostate cancer. At 2 years after treatment, more than 2 out of 5 patients are progression-free.

*Trial registration* Retrospectively registered.

## Background

Prostate cancer is the second most frequently diagnosed cancer in men worldwide and a relevant proportion of patients develop metastases during their disease course [1]. The standard-of-care for metastatic prostate cancer is palliative systemic therapy with androgen deprivation therapy (ADT) and/or chemotherapy [2]. Due to sensitive prostate specific antigen (PSA)-detection and improvements in imaging, metastatic disease is diagnosed more often at a time, when there are only a limited number of metastases [3]. Hellman and Weichselbaum 1995 introduced the term oligometastatic disease for a distinct cancer state between localized and widespread metastatic disease [4]. Recently, randomized phase II trials have demonstrated benefits of metastasis-directed therapy (MDT) in oligometastatic disease: The SABR-COMET study showed improved survival of stereotactic body radiotherapy (SBRT) when used in addition to standard-of-care systemic therapy [5], and two other trials demonstrated improved progression-free survival (PFS) in oligometastatic prostate cancer treated with MDT using radiotherapy compared to surveillance [6, 7]. However, many experts consider it necessary to further differentiate between different states of oligometastatic diseases, but there are still inconsistencies in the precise definition and nomenclature of these various subcategories [3, 8] [3, 8]. A recent European Society for Radiotherapy and Oncology (ESTRO) and European Organisation for Research and Treatment of Cancer (EORTC) consensus recommendation suggested a classification of oligometastatic disease depending on (1) genuine oligometastatic disease (patients without a history of polymetastatic disease) versus induced oligometastatic disease (patients with a history of polymetastatic disease), (2) de-novo oligometastatic disease (patients without a previous diagnosis of oligometastatic disease) versus repeat oligometastatic disease ﻿(patients with a previous diagnosis of oligometastatic disease), (3) synchronous oligometastatic disease (maximum 6 months interval between diagnosis of oligometastases and primary cancer diagnosis) versus metachronous oligometastatic disease (more than 6 months interval between diagnosis of oligometastases and primary cancer diagnosis), (4) development of oligometastatic disease in a treatment-free interval or during active systemic therapy and (5) oligopersistence (stable disease or partial response on current imaging) versus oligoprogression (progressive disease on current imaging) [9].

Prostate cancer patients with bone metastases have a worse prognosis than patients with distant lymph node metastases [10–12], and data describing the outcome of MDT in bone oligometastatic disease are sparse compared to data of MDT for lymph node metastases [13]. The aim of this work was to investigate the outcome of metastasis directed radiotherapy in prostate cancer patients with bone metastases with a focus on different categories of oligometastasis.

## Methods

### Patient selection and classification of oligometastatic disease

Consecutive oligometastatic prostate cancer patients treated with MDT to bone metastases between November 2011 and December 2019 at the University Hospital LMU Munich were retrospectively analyzed. Men with histologically confirmed prostate cancer who received definitive-intent radiotherapy to five or less bone metastases were eligible for this study [13]. Both castration sensitive and castration resistant patients were included, and patients were allowed to have concomitant ADT administered during MDT. All cases were discussed and approved by the multidisciplinary urooncologic tumor board. Individuals without any follow-up data, either in form of PSA or repeat imaging, were excluded from analysis. Based on the classification proposed by ESTRO and EORTC consensus recommendation [9], we classified oligometastatic disease as follows: First, a distinction was made between genuine oligometastatic disease and induced oligometastatic disease. Further, between de-novo and repeated disease. Patients with an interval less than 6 months between primary prostate cancer diagnosis and diagnosis of oligometastatic disease were considered to have synchronous oligometastatic disease. Metachronous disease was defined as an interval more than 6 months. Diagnosis of metachronous oligometastatic disease with the patient under active systemic therapy was considered metachronous oligoprogression. ﻿Bone lesion localization was categorized as follows: skull, ribs/clavicles/sternum, spine, pelvis including sacral bone and the extremities.

### Treatment characteristics

All patients underwent computed tomography (CT)-based treatment planning in supine position. The diagnostic imaging was co-registered for contouring. The planning target volume (PTV) comprised the macroscopic bony lesion with a margin depending on site and expected intrafractional motion. All patients received SBRT or conventionally fractionated intensity-modulated radiotherapy (IMRT) and image-guided radiotherapy (IGRT). The exact dose prescription depended on the volume and the localization of the lesion. Patients with synchronous oligometastatic disease and untreated primary received definitive radiotherapy to the primary simultaneously to MDT for oligometastasis. Patients diagnosed with local recurrence and/or pelvic lymph node-recurrence additional to bone metastases were treated simultaneously with radiotherapy to prostate fossa with or without whole-pelvic radiotherapy and boost to affected lymph nodes.

### Follow-up

PSA analysis was conducted every three to 6 months after radiotherapy. Biochemical failure was defined as (1) in the case of patients previously not treated with radical prostatectomy, the first PSA increase that was 2 ng/ml above the nadir after MDT; or (2) in the case of patients previously treated with radical prostatectomy, a PSA increase 0,2 ng/m above the nadir after MDT. Staging imaging was repeated if warranted by symptoms or change in PSA dynamics. Clinical failure was defined as progressive disease in imaging or initiation or escalation of systemic therapies. The decision regarding changes to a patient’s treatment paradigm and the initiation or escalation of the systemic therapy after progression was at the discretion of the treating urologist.

### Statistical analysis

All statistical analyses were conducted using SPSS Version 26 (IBM Corp., Armonk, NY, USA). Toxicity was graded according to the Common Terminology Criteria for Adverse Events (CTCAE) version 5.0. Acute toxicity was defined as toxicity occurring during RT or within 3 months thereafter. Endpoints evaluated were local control (LC), biochemical progression-free survival (bPFS) and clinical progression-free survival (cPFS). Time to progression was defined as time between the first date of MDT to the date of the first biochemical or clinical evidence of progression. Local failure was defined as tumor growth or metabolic increase of a treated lesion in re-staging imaging, in conjunction with a rising PSA value, in a previously stable lesion. Events of interest were PSA failure for bPFS and local or distant failure for cPFS. Survival analysis ﻿was performed using the Kaplan–Meier method. Univariate analysis using the log-rank test was conducted to evaluate the effect of age, Gleason score (GS), initial clinical or pathological T and N stage, PSA-doubling time (PSA-DT), number of metastases, hormone-sensitivity, category of oligometastatic disease, use of concomitant ADT and biologically effective dose (BED) as potential prognostic factors for bPFS and cPFS. Multivariate analysis was performed using the Cox proportional hazards model, using covariates with a p-value < 0.10 in univariate analysis. ﻿All p-values < 0.05 were considered statistically significant.

## Results

### Patient and treatment characteristics

Eighty prostate cancer patients with oligometastatic disease underwent MDT for bone metastases. Table 1 shows patient characteristics. Median age at diagnosis of oligometastases was 72 years (range 50–87 years). In the majority of cases, the initial T-stage was T3, and the initial N-stage was N0. Half of the patients (50%) had a Gleason score ≥ 9. Most patients’ initial treatment was radical prostatectomy (80.1%), and 31.3% subsequently had radiotherapy due to locoregional recurrence. Initial treatment was definitive radiotherapy, primary systemic therapy and no pretreatment in 5.0%, 11.3% and 3.8%, respectively. The median time between primary treatment and diagnosis of oligometastates was 22.1 months (range 0–260). At the time of MDT 31.3% of patients had de-novo synchronous oligometastatic disease, 46.3% had de-novo metachronous oligorecurrence after primary treatment and 22.5% had either de-novo oligoprogressive disease, repeat oligometastatic disease or induced oligometastatic disease. Forty-three patients (53.8%) had already ADT at some point during their disease course, 18 patients (22.5%) had CRPC.

**Table 1:** Patient characteristics

Patients, n	80
Age at MDT (years), median (range)	72 (50–87)
Initial tumor stage, n (%)	
T1	3 (3.8%)
T2	21 (26.3%)
T3	54 (67.5%)
Unknown	2 (2.5)
Initial nodal stage, n (%)	
N0	51 (63.7%)
N +	21 (26.3%)
Nx	5 (6.3%)
Unknown	3 (3.8%)
Gleason score, n (%)	
≤ 6	1 (1.3%)
7	26 (32.5%)
8	13 (16,3%)
≥ 9	40 (50.0%)
Initial PSA (ng/ml), median (range)	11,7 (3,6–1252)
Primary treatment, n (%)	
Surgery	39 (48.8%)
Surgery + postoperative RT	25 (31.3%)
Definitive RT	4 (5.0%)
Primary ADT or chemotherapy	9 (11.3%)
No pretreatment	3 (3.8%)
Time from primary treatment to MDT (months), median (range)	22.1 (0–260)
Category of oligometastatic disease, n (%)	
De novo synchronous oligometastatic disease	25 (31.3%)
Metachronous oligorecurrent disease	37 (46.3%)
Metachronous oligoprogressive disease	3 (3.8%)
Induced oligometastatic disease	7 (8.8%)
Repeat oligometastatic disease	8 (10.0%)
Previous ADT, n (%)	43 (53.8%)
HSPC/CRPC, n (%)	62 (77.5%)/18 (22.5%)

ADT, androgen deprivation therapy; CRPC, castration-resistant prostate cancer; HSPC, hormone-sensitive prostate cancer; MDT, metastasis-directed therapy; PSA, prostate specific antigen; RT, radiotherapy

Table 2 shows treatment characteristics*.* The staging method before diagnosis of OM was prostate specific membrane antigen—positron emission tomography/computed tomography (PSMA-PET/CT) in most cases (91.3%). Median PSA at time of imaging was 1.24 ng/ml (range 0.07–1252 ng/ml). Initially, 115 bone lesions were treated. The median number of metastases was one (range one–four) and 53 patients (66.3%) had a single metastasis treated. Sites of metastases were skull, thoracic, spine, pelvis and extremities in 0.7%, 45.2%, 10.4%, 39.1% and 3.5%, respectively. Median PSA before MDT was 1.23 ng/ml (range 0.07–1252). Data for PSA-DT before MDT was available for 49 patients and median pre-RT DT was 4.1 month.

**Table 2:** Treatment characteristics

Imaging method prior to MDT, n (%)	
PSMA-PET/CT	73 (91.3%)
Choline-PET/CT	3 (3.8%)
Bone scan with or without CT/MRI, n (%)	4 (5.0%)
PSA at imaging (ng/ml), median (range)	1.24 (0.07–1252)
Number of metastases, n (%)	
One metastasis	53 (66.3%)
Two metastases	20 (25.0%)
Three metastases	6 (7.5%)
Four metastases	1 (1.3%)
Metastatic site, number of lesions (%)	
Skull	2 (0.7%)
Thoracic (ribs/clavicles/sternum)	52 (45.2%)
Spine	12 (10.4%)
Pelvis including sacral bone	45 (39.1%)
Extremities	4 (3.5%)
Type of treatment, n (%)	
MDT alone	49 (61.3%)
MDT + salvage RT to prostate bed and/or local recurrence and/or pelvis and regional LN	21 (26.3%)
MDT + definitive RT to primary	10 (12.5%)
Pre-MDT PSA (ng/ml), median (range)	1.23 (0.07–1252)
Pre-MDT PSA-DT (month), median (range)	4.1 (0.7–34.0)
Dose prescription	
40 Gy/10 fractions (BED_3_: 93.3 Gy)	n = 45 (56.3%)
30 Gy/5 fractions (BED_3_: 90 Gy)	n = 17 (21.3%)
50.4 Gy/28 fractions (BED_3_: 80.6 Gy)	n = 6 (7.5%)
56 Gy/28 fractions (BED_3_: 93.3 Gy)	n = 3 (3.8%)
35 Gy/10 fractions (BED_3_: 75.8 Gy)	n = 2 (2.5%)
50 Gy/25 fractions (BED_3_: 83.3 Gy)	n = 2 (2.5%)
55 Gy/25 fractions (BED_3_: 95.3 Gy)	n = 1 (1.3%)
52,8 Gy/32 fractions (BED_3_: 81.8 Gy)	n = 1 (1.3%)
50 Gy/20 fractions (BED_3_: 91.7 Gy)	n = 1 (1.3%)
42 Gy/14 fractions (BED_3_: 84.0 Gy)	n = 1 (1.3%)
39 Gy/13 fractions (BED_3_: 78.0 Gy)	n = 1 (1.3%)
Concomitant ADT, n (%)	55 (68.8%)

ADT, androgen deprivation therapy; BED, biologically effective dose; CT, computed tomography; MDT, metastasis-directed therapy; MRI, magnetic resonance imaging; PET, positron emission tomography; PSA, prostate specific antigen; PSA-DT, PSA-doubling time; PSMA, prostate specific membrane antigen; RT, radiotherapy

The median BED using an α/β value of 3 was 93.3 Gy (range 75.8–95.3 Gy), and the most common fractionation schemes were 40 Gy in 10 fractions (56.3%) and 30 Gy in 5 fractions (21.3%). Fifty-five patients (68.8%) received concomitant ADT.

### Clinical outcomes

**Fig. 1:**
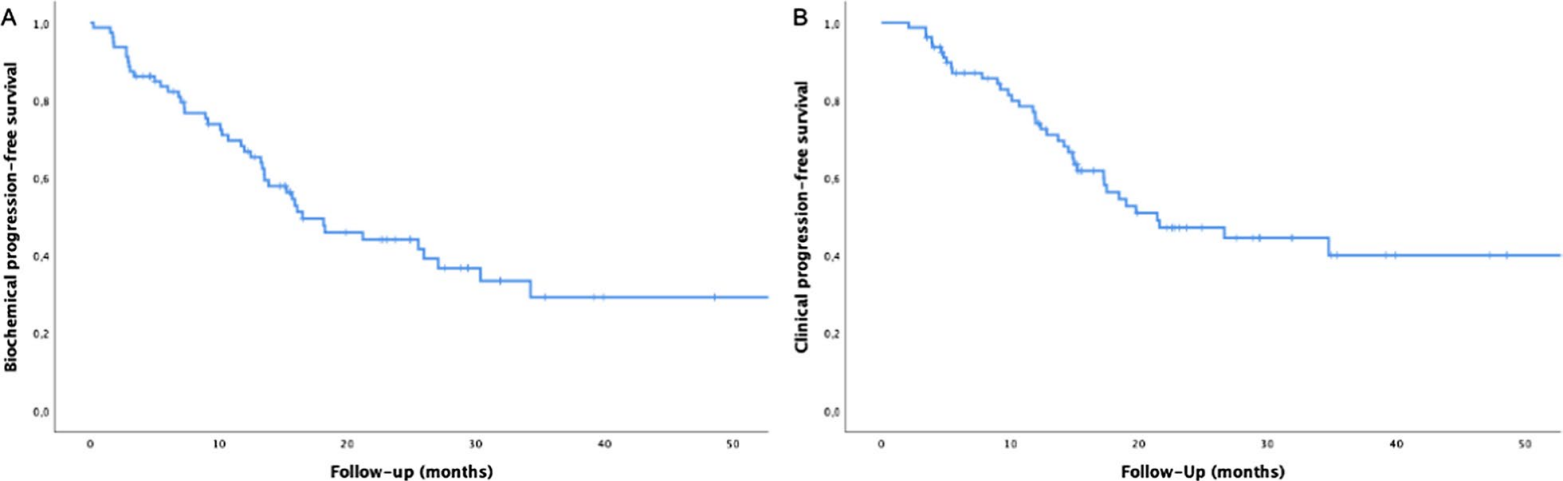
Kaplan–Meier curves: biochemical progression-free survival (**a**), clinical progression-free survival (**b**)

On first follow-up, 64 patients (80%) had PSA stability or decline after MDT and reached the PSA nadir after a median time of 3.4 months. Of 25 patients without concomitant ADT, PSA declined or was stable after RT in 14 patients (56.0%). After a median follow-up time of 23 months (range 4–99 months) 45 patients (56.2%) had biochemical recurrence. Median bPFS was 16.5 months (95% confidence interval [CI] 11.3–22.7 months) and estimated bPFS at 1 and 2 years were 66.8% and 44.1%, respectively (Fig. 1a). In patients with stable or declined PSA-value median bPFS was 29.6 months (95% confidence interval [CI] 16.0–35.9 months). Median cPFS in the whole population was 21.5 months (95% confidence interval [CI] 11.9–31.0 months) and estimated cPFS at 1 and 2 years were 74.1% and 47.2%, respectively (Fig. 1b). In patients with stable or declined PSA-value median cPFS was 34.8 months (95% confidence interval [CI] 17.6–51.9 months). The local failure rate at 12 and 24 months were 0% and 1.7%, respectively.

**Fig. 2:**
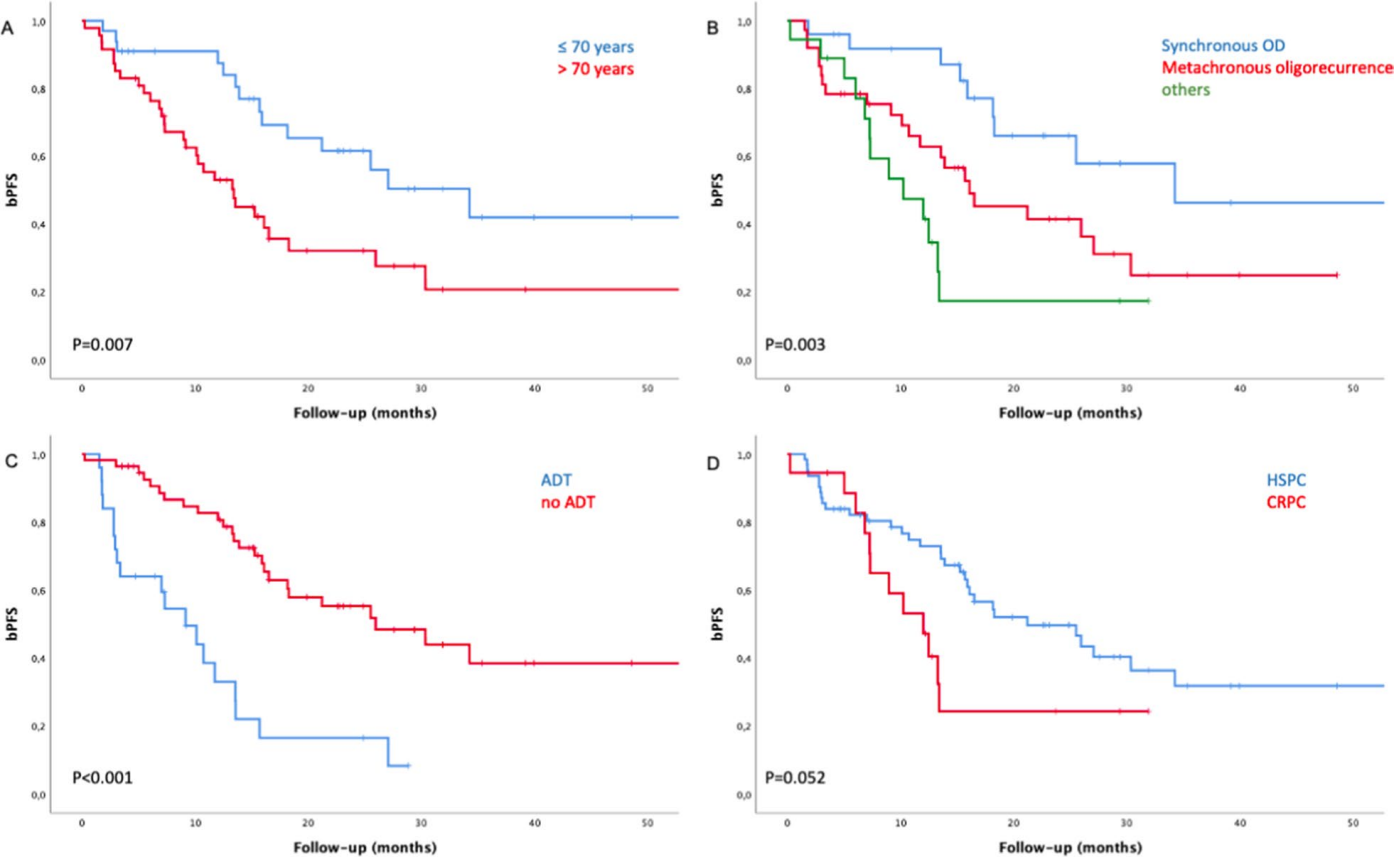
Kaplan–Meier curves: biochemical progression-free survival in patients aged ≤ 70 and > 70 years (**a**), with synchronous oligometastatic disease, metachronous oligorecurrent disease or other (de-novo oligoprogressive, repeat oligometastatic or induced oligometastatic disease) (**b**), with or without concomitant ADT (**c**), with hormone-sensitive prostate cancer or castration-resistant prostate cancer (**d**)

Tables 3 and 4 show the results of uni- and multivariate analysis. Factors predictive for bPFS in univariate analysis were age, category of oligometastatic disease and concomitant ADT (Fig. 2a–c). There was a trend for better bPFS for patients with hormone-sensitive prostate cancer (HSPC) compared to patients with castration-resistant prostate cancer (CRPC) (P = 0.052) (Fig. 2d). Patients aged 70 or younger had a median bPFS of 34.3 months compared with 13.7 months in patients older 70 years (P = 0.007). Patients with de-novo synchronous oligometastatic disease had a median bPFS of 34.3 months compared with 16.1 months for de-novo oligorecurrent and 10.3 months for the group with oligoprogressive, repeat oligometastatic or induced oligometastatic disease, (P = 0.003). Median bPFS was 26.0 vs. 9.2 months in patients treated with and without concomitant ADT (P < 0.001). On multivariate analysis age and concomitant ADT stayed significant for bPFS. Factors predictive for cPFS in univariate analysis were age, hormone-sensitivity, category of oligometastatic disease and concomitant ADT. Once again, younger age and concomitant ADT were significantly associated with improved cPFS.

**Table 3:** univariate analysis of prognostic factors for bPFS and cPFS

Patient characteristics	n	Median bPFS (months)	p-value	Median cPFS (months)	p-value
Age at diagnosis of oligometastases			**0.007**		**0.008**
≤ 70 years	33	34.3		Not reached	
> 70 years	47	13.4		15.0	
Gleason score			0.622		0.877
≤ 8	40	18.3		21.6	
≥ 9	40	15.9		18.5	
Initial tumor stage			0.710		0.601
≤ T2	24	18.3		21.6	
≥ T3	54	16.5		21.5	
Initial nodal stage			0.658		0.227
N0	51	15.9		17.5	
N1	21	21.2		80.1	
PSA-DT			0.928		0.952
≤ 4 months	24	15.9		19.0	
> 4 months	25	13.6		15.2	
Number of metastases			0.471		0.413
1	53	16.1		18.5	
≥ 2	27	16.5		34.8	
Hormone-sensitivity			0.052		**0.040**
HSPC	62	21.2		26.5	
CRPC	18	12.0		12.8	
Category of oligometastatic disease			**0.003**		**0.003**
De-novo synchronous oligometastatic disease	25	34.3		80.9	
De-novo metachronous oligorecurrent disease	37	16.1		19.0	
Other (de-novo oligoprogressive, repeat oligometastatic or induced oligometastatic disease)	18	10.3		12.4	
Concomitant ADT			**0.000007**		**0.000149**
Present	55	26.0		34.8	
Absent	25	9.2		12.4	
Dose BED_3_			0.142		0.102
≤ 93 Gy	31	26.0		17.3	
> 93 Gy	49	13.9		80.9	

ADT, androgen deprivation therapy; BED, biologically effective dose; bPFS, biochemical progression-free survival; cPFS, clinical progression-free survival; CRPC, castration-resistant prostate cancer; HSPC, hormone-sensitive prostate cancer; PSA, prostate specific antigen; PSA-DT, PSA-doubling time

**Table 4:** Multivariate analysis of predictive factors for bPFS and cPFS

Patient characteristics	bPFS		cPFS	
	p-value	HR (95% CI)	p-value	HR (95% CI)
Age at diagnosis of OD	**0.007**	2.69 (1.32–5.51)	**0.015**	2.60 (1.20–5.62)
Hormone-sensitivity	0.911	1.08 (0.29–4.04)	0.945	0.95 (0.23–3.93)
Category of OD	0.113	1.91 (0.86–4.24)	0.096	2.14 (0.87–5.27)
Concomitant ADT	**0.000044**	0.21 (0.10–0.44)	**0.001**	0.26 (0.12–0.58)

ADT, androgen deprivation therapy; bPFS, biochemical progression-free survival; cPFS, clinical progression-free survival; HR, hazard ratio; OD, oligometastatic disease

MDT was well tolerated. No patient experienced grade three or higher toxicity. Acute grade two toxicity was observed in five patients (6.3%) consisting of diarrhoea and bone pain. Four patients (5%) had late grade two toxicity consisting of proctitis, erectile dysfunction and consisting bone pain. Of note, three of these four patients had simultaneously a definitive RT to the primary, which could have caused or at least contributed to the adverse effects. No patient experienced a compression fracture of vertebra.

### Pattern of progression and repeated MDT

Table 5 gives an overview of the pattern of progression and the following therapies. Progression after MDT was local failure, new bone metastases, lymph node metastases, local recurrence in the prostate fossa and biochemical relapse only in 4.4%, 60.0%, 17.7%, 6.6% and 13.3%, respectively. Of the patients who progressed with new bone metastases, eight patients had polymetastatic relapse (> five metastases) and 19 patients had repeat oligometastatic disease with median two new metastases (range one–five). Thirteen patients with repeat oligometastatic disease underwent repeated MDT for 21 bone lesions. Median bPFS and cPFS after this second course of MDT were 13.1 and 14.0 months, respectively. Two patients received a third course of MDT without additional toxicity.

**Table 5:** Pattern of progression following MDT

Metastatic location	n (%)	Therapy	n (%)
Local failure	2 (4.4%)	ADT initiation/escalation	2 (4.4%)
≤ 5 new bone metastases	19 (42.2%)	Repeated MDT	10 (22.2%)
		ADT initiation/escalation	6 (13.3%)
		Repeated MDT + ADT initiation/escalation	3 (6.6%)
> 5 new bone metastases	8 (17.7%)	ADT initiation/escalation	5 (11.1%)
		Lutetium radio-ligand therapy	2 (4.4%)
		None	1 (2.2%)
Lymph node recurrence	8 (17.7%)	Salvage-RT + ADT initiation/escalation	4 (8.8%)
		ADT escalation	3 (6.6%)
		None	1 (2.2%)
Prostate fossa recurrence	3 (6.6%)	Salvage-RT + ADT initiation/escalation	2 (4.4%)
		ADT initiation	1 (2.2%)
Visceral metastases	2 (4.4%)	Chemotherapy initiation	1 (2.2%)
		Repeated MDT	1 (2.2%)
Biochemical progression only	7 (13.3%)	ADT initiation/escalation	3 (6.6%)
		None	4 (8.8%)

ADT, androgen deprivation therapy; MDT, metastasis-directed therapy; PSA, prostate specific antigen; RT, radiotherapy

## Discussion

To our knowledge, this study, including data from 80 patients, is the largest series of patients with oligometastatic prostate cancer treated with MDT for bone metastases. We could demonstrate a high LC and a favorable toxicity profile. After 2 years, more than two out of five patients were free of progression. Age ≤ 70 years and concomitant ADT were predictive factors for improved bPFS and cPFS.

The observed median bPFS and cPFS of 16 and 21 months, respectively, are comparable to the results of MDT for bone metastases in literature [14–16]. Bone metastases are considered to have a worse prognosis compared to lymph node metastases in metastatic prostate cancer [11], and the majority of experts in the advanced prostate cancer consensus conference (APCCC) voted for the distinction of these two kinds of metastatic patterns [8]. As all included patients in our study had bone oligometastases, no direct comparison with patients with lymph node oligometastases was possible. However, our results are also similar to data in literature of MDT for lymph node metastases with median PFS ranging from 15 to 18 months [17–20].

The best therapeutic strategy for patients with local recurrence in the prostate fossa or pelvic lymph node recurrence after radical prostatectomy is still under debate. Retrospective data show good results for salvage radiotherapy in this situation [21–24]. Twenty-one patients in our study presented with bone oligometastatic disease and concurrent local recurrence in the prostate bed and/or pelvic lymph node recurrence. Leaving these potential sources of tumor spread untreated, would have foiled the effect of MDT. Thus, a therapeutic approach with radiotherapy to all macroscopic disease was chosen for these patients, i.e. MDT plus radiotherapy to prostate fossa with or without whole-pelvic radiotherapy and boost to affected lymph nodes.

PSMA-PET/CT is increasingly finding its way into clinical practice and a high sensitivity and specificity for detection of bone metastases are reported [25–29]. In our series the vast majority (91%) of patients was staged with PSMA-PET/CT before MDT. However, a relevant proportion (20%) had rising PSA levels after MDT, indicating that subclinical and thus untreated disease was present at the time of imaging. Obviously, the proportion was even higher in patients without concomitant ADT (44%). This underscores the importance of additional indicators to distinguish in advance the patients with an occult polymetastatic state from those with “true” oligometastatic disease.

No consensus exists on the concomitant use of systemic therapy in oligometastatic prostate cancer. On the one hand ADT and its combination therapies can be associated with significant side effects and deterioration of the quality of life. Therefore, some experts aim to defer systemic therapies by MDT. Androgen deprivation therapy-free survival (ADT-FS) was introduced as a new endpoint and reported by some authors, ranging between 7 and 66 months with MDT [13]. Furthermore, the STOMP randomized phase II trial showed a prolonged ADT-FS compared to surveillance (21 vs. 13 months) [6]. On the other hand, there is a clear body of evidence showing improved overall survival with systemic therapies in metastatic disease [30–32]. In our series, concomitant ADT not only was a predictor of improved bPFS in multivariate analysis but also of improved cPFS. Given that subclinical disease is missed by imaging in a significant proportion of oligometastatic patients, concomitant ADT may contribute to long-term tumor control. Therefore, omitting ADT may be correlated with a decreased survival while temporarily delaying its side effects. This point should be considered and discussed in the context of informed decision-making with patients. Indeed, the majority of panelists of APCCC voted for adding MDT to systemic therapies, instead of replacing them [8].

Of those patients with disease progression, almost one-third relapsed in an oligometastatic pattern and received a second course of MDT, and two patients underwent a third course of MDT without increased toxicity. This is in accordance with previous reports, which reported second, third and fourth courses of MDT with favorable toxicity profiles.

In our series, MDT resulted in a very good LC of 98.3% at 2 years, compared to the results of other published studies, ranging from 75 to 100% [14–16, 33–35]. This was despite the fact that, compared to radiotherapy schedules in other studies, a relative low BED_3_ with median 93.3 Gy (range 75.8–95.3 Gy) was used. Ost et al. found improved LC of MDT at BED_3_ > 100 Gy and Hurmuz and colleagues reported improved PFS at BED_3_ > 108 Gy [36, 37]. In our study, no dose effect on bPFS or cPFS was found. Of note, in the aforementioned series both lymph node and bone metastases were treated and the majority of bone lesions were in the spine. In contrast, most lesions in our series were non-spine metastases (89.4%). In a recent study of international practice patterns of SBRT for non-spine metastases all experts agreed on dose regimens with a BED ≤ 100 Gy [38]. However, due to the limited patient number and follow-up time, our results regarding local control should be taken with caution.

The majority of panelists of APCCC agreed that different categories of oligometastases need to be distinguished [39]. Many studies of MDT in prostate cancer, including the two phase II trials, included patients with metachronous oligorecurrent disease, but not with synchronous oligometastases [6, 7]. The randomized phase III STAMPEDE trial showed that primary directed therapy (PDT) improved failure-free survival in patients with synchronous metastatic disease with a low metastatic burden, but MDT was not performed [40]. We divided the included patients into different categories of oligometastatic disease according to the recent ESTRO/EORTC recommendation. However, there were few patients within the categories metachronous oligoprogressive disease, induced oligometastatic disease and recurrent oligometastatic disease, so we grouped them together for univariate and multivariate analysis. In our series, bPFS was more than twice as long and cPFS was more than four times longer in patients with synchronous metastatic disease compared with patients with de novo oligorecurrent oligometastatic disease. This appears to contradict the findings of other retrospective data, that reported improved oncologic outcome with increasing interval between therapy of primary and diagnosis of oligometastases [41–43].

One possible reason for this could be that several definitions for synchronous oligometastatic disease are available in the literature. No consensus exists on the exact interval between the diagnosis of the primary tumor and the detection of oligometastases to distinguish between metachronous and occult synchronous disease. Some studies define oligometastatic disease as synchronous only if oligometastases are detected at initial staging. Current guidelines recommend staging with bone scan before primary therapy depending on risk factors such as Gleason score, and staging with PET/CT is recommended only in cases with biochemical recurrence, although PET/CT has a higher detection rate for bone metastases compared with bone scan [44, 45]. This may result in synchronous oligometastatic disease being diagnosed rather in those patients who have high-risk factors for aggressive tumor growth (and thus have initial staging) and in those patients with a more extensive (and therefore detectable with bone scan) oligometastatic disease. However, another commonly used definition of synchronous disease, which we applied, is an interval of less than 6 months between primary prostate cancer diagnosis and diagnosis of oligometastatic disease. This definition results in more patients with low-risk profile and initial low metastatic burden categorized as synchronous oligometastatic disease, and subsequently could lead to an improved outcome of these category.

Patients with HSPC had significant longer cPFS in univariate analysis compared to patients with CRPC. This is in accordance with data from previous studies, showing worse PFS in oligometastatic patients with CRPC [46–49]. On the other hand, Berghen and colleagues showed benefits of MDT in patients with CRPC in terms of postponement of next-line systemic treatment [50].

Our study has some limitations due to its retrospective and monocentric nature. Furthermore, as the median follow-up time is relatively short the relevant endpoints overall survival or prostate cancer specific survival could not be assessed. Pretreatment imaging in the majority of patients was PSMA PET/CT, which has been shown to have high specificity in detecting prostate cancer metastases [25]. However, because histologic verification was not performed, we could not exclude false-positive and false-negative PSMA PET lesions. The use of concomitant ADT was inconsistent, which complicated the interpretation of PSA kinetics and bPFS.

However, until further prospective data is available, this study sheds some light on the previously underrepresented group of patients with bone oligometastatic prostate cancer treated with MDT and stratified by current ESTRO/EORTC subclassifications for oligometastatic disease. Although hormone-sensitivity and the category of oligometastatic disease were no longer significant factors in the multivariate analysis, the large differences in the oncological outcome between the groups still highlight the need for a finer differentiation of patients with bone oligometastatic disease.

## Conclusions

MDT for bone oligometastatic prostate cancer is safe and offers high local control rates. At 2 years after treatment, more than 2 out of 5 patients are free from progression. Further subclassification of oligometastatic disease seems reasonable to identify the patients, which benefit the most from MDT.

## Data Availability

Research data are stored in an institutional repository and will be shared upon request to the corresponding author.

## Abbreviations

ADT: Androgen deprivation therapy
ADT-FS: Androgen deprivation therapy-free survival
APCCC: Advanced prostate cancer consensus conference
BED: Biologically effective dose
bPFS: Biochemical progression-free survival
CI: Confidence interval
cPFS: Clinical progression-free survival
CRPC: Castration-resistant prostate cancer
CT: Computed tomography
CTCAE: Common Terminology Criteria for Adverse Events
ESTRO: European Society for Radiotherapy and Oncology
EORTC: European Organisation for Research and Treatment of Cancer
GS: Gleason score
HR: Hazard ratio
HSPC: Hormone-sensitive prostate cancer
IGRT: Image-guided radiotherapy
IMRT: Intensity-modulated radiotherapy
LC: Local control
MDT: Metastasis-directed therapy
MRI: Magnetic resonance imaging
OD: Oligometastatic disease
PDT: Primary-directed therapy
PET/CT: Positron emission tomography/computed tomography
PFS: Progression-free survival
PSA: Prostate specific antigen
PSA-DT: PSA-doubling time
PSMA: Prostate specific membrane antigen
PTV: Planning target volume
RT: Radiotherapy
SBRT: Stereotactic body radiotherapy

## References

[1] Bray F, Ferlay J, Soerjomataram I, Siegel RL, Torre LA, Jemal A (2018). Global cancer statistics 2018: GLOBOCAN estimates of incidence and mortality worldwide for 36 cancers in 185 countries. CA Cancer J Clin.

[2] Cornford P, Bellmunt J, Bolla M (2017). EAU-ESTRO-SIOG guidelines on prostate cancer. Part II: treatment of relapsing, metastatic, and castration-resistant prostate cancer. Eur Urol.

[3] Lievens Y, Guckenberger M, Gomez D (2020). Defining oligometastatic disease from a radiation oncology perspective: an ESTRO-ASTRO consensus document. Radiother Oncol.

[4] Hellman W (1995). Oligometastases.

[5] Palma DA, Olson R, Harrow S (2019). Stereotactic ablative radiotherapy versus standard of care palliative treatment in patients with oligometastatic cancers (SABR-COMET): a randomised, phase 2, open-label trial. Lancet.

[6] Ost P, Reynders D, Decaestecker K (2017). Surveillance or metastasis-directed therapy for oligometastatic prostate cancer recurrence: a prospective, randomized, multicenter phase II trial. J Clin Oncol.

[7] Phillips R, Shi WY, Deek M (2020). Outcomes of observation vs stereotactic ablative radiation for oligometastatic prostate cancer: the ORIOLE phase 2 randomized clinical trial. JAMA Oncol.

[8] Gillessen S, Attard G, Beer TM (2020). Management of patients with advanced prostate cancer: report of the advanced prostate cancer consensus conference 2019 [formula presented]. Eur Urol.

[9] Guckenberger M, Lievens Y, Bouma AB (2020). Characterisation and classification of oligometastatic disease: a European Society for Radiotherapy and Oncology and European Organisation for Research and Treatment of Cancer consensus recommendation. Lancet Oncol.

[10] Liu D, Kuai Y, Zhu R (2020). Prognosis of prostate cancer and bone metastasis pattern of patients: a SEER-based study and a local hospital based study from China. Sci Rep.

[11] Halabi S, Kelly WK, Ma H (2016). Meta-analysis evaluating the impact of site of metastasis on overall survival in men with castration-resistant prostate cancer. J Clin Oncol.

[12] Guo Y, Mao S, Zhang A (2019). Prognostic significance of young age and non-bone metastasis at diagnosis in patients with metastatic prostate cancer: a SEER population-based data analysis. J Cancer.

[13] Rogowski P, Roach M, Schmidt-Hegemann NS (2021). Radiotherapy of oligometastatic prostate cancer: a systematic review. Radiat Oncol.

[14] Patel PH, Lee C, Alison C, Mansour CT, Nicholas S (2019). Stereotactic body radiotherapy for bone oligometastatic disease in prostate cancer. World J Urol.

[15] Siva S, Bressel M, Murphy DG (2018). Stereotactic abative body radiotherapy (SABR) for oligometastatic prostate cancer: a prospective clinical trial. Eur Urol.

[16] Muldermans JL, Romak LB, Kwon ED, Park SS, Olivier KR (2016). Stereotactic body radiation therapy for oligometastatic prostate cancer. Int J Radiat Oncol Biol Phys.

[17] Jereczek-Fossa BA, Fanetti G, Fodor C (2017). Salvage stereotactic body radiotherapy for isolated lymph node recurrent prostate cancer: single institution series of 94 consecutive patients and 124 lymph nodes. Clin Genitourin Cancer.

[18] Triggiani L, Alongi F, Buglione M (2017). Efficacy of stereotactic body radiotherapy in oligorecurrent and in oligoprogressive prostate cancer: New evidence from a multicentric study. Br J Cancer.

[19] Nicosia L, Franzese C, Mazzola R (2020). Recurrence pattern of stereotactic body radiotherapy in oligometastatic prostate cancer: a multi-institutional analysis. Strahlentherapie Onkol.

[20] Schmidt-Hegemann NS, Buchner A, Eze C (2020). PSMA-positive nodal recurrence in prostate cancer: salvage radiotherapy is superior to salvage lymph node dissection in retrospective analysis. Strahlentherapie Onkol.

[21] Trock BJ, Han M, Freedland SJ (2008). Prostate cancer-specific survival following salvage radiotherapy vs observation after radical prostatectomy. JAMA J Am Med Assoc.

[22] van Stam MA, Aaronson NK, Pos FJ (2016). The effect of salvage radiotherapy and its timing on the health-related quality of life of prostate cancer patients. Eur Urol.

[23] Fodor A, Lancia A, Ceci F (2018). Oligorecurrent prostate cancer limited to lymph nodes: getting our ducks in a row: nodal oligorecurrent prostate cancer. World J Urol.

[24] Tran S, Jorcano S, Falco T, Lamanna G, Miralbell R, Zilli T (2018). Oligorecurrent nodal prostate cancer. Am J Clin Oncol Cancer Clin Trials.

[25] Zacho HD, Nielsen JB, Haberkorn U, Stenholt L, Petersen LJ (2018). 68Ga-PSMA PET/CT for the detection of bone metastases in prostate cancer: a systematic review of the published literature. Clin Physiol Funct Imaging.

[26] Unterrainer M, Eze C, Ilhan H (2020). Recent advances of PET imaging in clinical radiation oncology. Radiat Oncol.

[27] Mazzola R, Francolini G, Triggiani L, et al. Metastasis-directed therapy (SBRT) guided by PET-CT 18F-CHOLINE versus PET-CT 68Ga-PSMA in castration-sensitive oligorecurrent prostate cancer: a comparative analysis of effectiveness. *Clin Genitourin Cancer*. 2020:1–7. 10.1016/j.clgc.2020.08.002 10.1016/j.clgc.2020.08.002 32863189

[28] Mazzola R, Cuccia F, Figlia V (2019). New metabolic tracers for detectable PSA levels in the postprostatectomy setting: Is the era of melting glaciers upcoming?. Transl Androl Urol.

[29] Mazzola R, Cuccia F, Figlia V (2021). Stereotactic body radiotherapy for oligometastatic castration sensitive prostate cancer using 15 T MRI-Linac: preliminary data on feasibility and acute patient-reported outcomes. Radiol Med.

[30] Fizazi K, Tran N, Fein L, et al. Abiraterone plus Prednisone in Metastatic, Castration-Sensitive Prostate Cancer. *N Engl J Med*. 2017:NEJMoa1704174. 10.1056/NEJMoa1704174 10.1056/NEJMoa1704174 28578607

[31] James ND, Sydes MR, Clarke NW (2016). Addition of docetaxel, zoledronic acid, or both to first-line long-term hormone therapy in prostate cancer (STAMPEDE): Survival results from an adaptive, multiarm, multistage, platform randomised controlled trial. Lancet.

[32] Kyriakopoulos CE, Chen YH, Carducci MA (2018). Chemohormonal therapy in metastatic hormone-sensitive prostate cancer: long-term survival analysis of the randomized phase III E3805 chaarted trial. J Clin Oncol.

[33] Habl G, Straube C, Schiller K (2017). Oligometastases from prostate cancer: Local treatment with stereotactic body radiotherapy (SBRT). BMC Cancer.

[34] Fanetti G, Marvaso G, Ciardo D (2018). Stereotactic body radiotherapy for castration-sensitive prostate cancer bone oligometastases. Med Oncol.

[35] Wu JX, Lin LM, He JY, Hong L, Li JL (2016). Radiotherapy combined with androgen deprivation for bone oligometastases after primary curative radiotherapy for prostate cancer. Med (United States).

[36] Ost P, Jereczek-Fossa BA, Van AN (2016). Progression-free survival following stereotactic body radiotherapy for oligometastatic prostate cancer treatment-naive recurrence: a multi-institutional analysis. Eur Urol.

[37] Hurmuz P, Onal C, Ozyigit G, et al. Treatment outcomes of metastasis-directed treatment using 68Ga-PSMA-PET/CT for oligometastatic or oligorecurrent prostate cancer: Turkish Society for Radiation Oncology group study (TROD 09-002). *Strahlentherapie Onkol*. 2020:1034–1043. doi:10.1007/s00066-020-01660-6 10.1007/s00066-020-01660-6 32617620

[38] Nguyen TK, Sahgal A, Dagan R (2020). Stereotactic body radiation therapy for nonspine bone metastases: international practice patterns to guide treatment planning. Pract Radiat Oncol.

[39] Gillessen S, Attard G, Beer TM (2018). Management of patients with advanced prostate cancer: the report of the advanced prostate cancer consensus conference APCCC 2017 [figure presented]. Eur Urol.

[40] Parker CC, James ND, Brawley CD (2018). Articles radiotherapy to the primary tumour for newly diagnosed, metastatic prostate cancer (STAMPEDE): a randomised controlled phase 3 trial. Lancet.

[41] Lépinoy A, Silva YE, Martin E, et al. Salvage extended field or involved field nodal irradiation in 18 F-fluorocholine PET/CT oligorecurrent nodal failures from prostate cancer. 2019:40–48. 10.1007/s00259-018-4159-0 30267117

[42] Ong WL, Koh TL, Lim Joon D (2019). Prostate-specific membrane antigen-positron emission tomography/computed tomography (PSMA-PET/CT)-guided stereotactic ablative body radiotherapy for oligometastatic prostate cancer: a single-institution experience and review of the published literature. BJU Int.

[43] Kalinauskaite G, Senger C, Kluge A (2020). 68Ga-PSMA-PET/CT-based radiosurgery and stereotactic body radiotherapy for oligometastatic prostate cancer. PLoS ONE.

[44] Registernummer A-. Interdisziplinäre Leitlinie der Qualität S3 zur Früherkennung , Diagnose und Therapie der ver-schiedenen Stadien des Prostatakarzinoms Wesentliche Neuerungen durch die Aktualisierung. 2019:1–345.

[45] Mottet N, Bellmunt J, Briers E, et al. Guidelines on Prostate Cancer. *Update*. 2020;53(February):31–45. http://www.uroweb.org/fileadmin/tx_eauguidelines/2005/Pocket/Prostate_Cancer.pdf.

[46] Franzese C, Agostino GD, Navarria P (2018). The efficacy of Stereotactic body radiation therapy and the impact of systemic treatments in oligometastatic patients from prostate cancer. Cancer Med.

[47] Guler OC, Engels B, Onal C (2018). The feasibility of prostate-specific membrane antigen positron emission tomography(PSMA PET/CT)-guided radiotherapy in oligometastatic prostate cancer patients. Clin Transl Oncol.

[48] Patel PH, Chaw CL, Tree AC, Sharabiani M, van As NJ (2019). Stereotactic body radiotherapy for bone oligometastatic disease in prostate cancer. World J Urol.

[49] Deek MP, Yu C, Phillips R (2019). Radiation therapy in the definitive management of oligometastatic prostate cancer: the Johns Hopkins experience. Int J Radiat Oncol Biol Phys.

[50] Berghen C, Joniau S, Ost P (2019). Progression-directed therapy for oligoprogression in castration-refractory prostate cancer. Eur Urol Oncol.

